# The mysterious orphans of *Mycoplasmataceae*

**DOI:** 10.1186/s13062-015-0104-3

**Published:** 2016-01-08

**Authors:** Tatiana V. Tatarinova, Inna Lysnyansky, Yuri V. Nikolsky, Alexander Bolshoy

**Affiliations:** Children’s Hospital Los Angeles, Keck School of Medicine, University of Southern California, Los Angeles, 90027 CA USA; Spatial Sciences Institute, University of Southern California, Los Angeles, 90089 CA USA; Mycoplasma Unit, Division of Avian and Aquatic Diseases, Kimron Veterinary Institute, POB 12, Beit Dagan, 50250 Israel; School of Systems Biology, George Mason University, 10900 University Blvd, MSN 5B3, Manassas, VA 20110 USA; Prosapia Genetics, LLC, 534 San Andres Dr., Solana Beach, CA 92075 USA; Vavilov Institute of General Genetics, Moscow, Russian Federation; Department of Evolutionary and Environmental Biology and Institute of Evolution, University of Haifa, Haifa, Israel

**Keywords:** *Mycoplasmataceae*, COG, ORFan, HHP, Evolution, Stop codon

## Abstract

**Background:**

The length of a protein sequence is largely determined by its function. In certain species, it may be also affected by additional factors, such as growth temperature or acidity. In 2002, it was shown that in the bacterium *Escherichia coli* and in the archaeon *Archaeoglobus fulgidus,* protein sequences with no homologs were, on average, shorter than those with homologs (BMC Evol Biol 2:20, 2002). It is now generally accepted that in bacterial and archaeal genomes the distributions of protein length are different between sequences with and without homologs. In this study, we examine this postulate by conducting a comprehensive analysis of all annotated prokaryotic genomes and by focusing on certain exceptions.

**Results:**

We compared the distribution of lengths of “having homologs proteins” (HHPs) and “non-having homologs proteins” (orphans or ORFans) in all currently completely sequenced and COG-annotated prokaryotic genomes. As expected, the HHPs and ORFans have strikingly different length distributions in almost all genomes. As previously established, the HHPs, indeed are, on average, longer than the ORFans, and the length distributions for the ORFans have a relatively narrow peak, in contrast to the HHPs, whose lengths spread over a wider range of values. However, about thirty genomes do not obey these rules. Practically all genomes of *Mycoplasma* and *Ureaplasma* have atypical ORFans distributions, with the mean lengths of ORFan larger than the mean lengths of HHPs. These genera constitute over 80 % of atypical genomes.

**Conclusions:**

We confirmed on a ubiquitous set of genomes that the previous observation of HHPs and ORFans have different gene length distributions. We also showed that *Mycoplasmataceae* genomes have very distinctive distributions of ORFans lengths. We offer several possible biological explanations of this phenomenon, such as an adaptation to *Mycoplasmataceae*’s ecological niche, specifically its “quiet” co-existence with host organisms, resulting in long ABC transporters.

**Electronic supplementary material:**

The online version of this article (doi:10.1186/s13062-015-0104-3) contains supplementary material, which is available to authorized users.

## Background

Different factors affect properties of prokaryotic proteins [1]. Some of them appear to be general constraints on protein evolution. For example, genomic studies revealed that the base composition of a genome (i.e. GC content) correlates with the overall amino acid composition of its proteins [2]. There are also general constraints on protein size, such as, in general, smaller proteins for prokaryotes compare to eukaryotes [3]. Previously, we revealed some other factors affecting the lengths of proteins having homologues in other genomes [4–6]. However, there are numerous protein-encoding genes without homologues in genomes of other organisms called “ORFans” or “orphans” (the term coined by Fisher and Eisenberg [7]). The ORFans are not linked by overall similarity or shared domains to the genes or gene families characterized in other organisms. Tautz and Domazet-Lošo [8] were the first to discuss systematic identification of ORFan genes in the context of gene emergence through duplication and rearrangement processes. Their study was supported by other excellent reviews [9–11].

ORFan genes were initially described in yeast as a finding of the yeast genome-sequencing project [12, 13], followed by identification of ORFans in all sequenced bacterial genomes. Comparative genomics has shown that ORFans are an universal feature of any genome, with a fraction of ORFan genes varying between 10 and 30 % per a bacterial genome [14]. Fukuchi and Nishikawa [15] identified that neither organism complexity nor the genome length correlate with the percentage of ORFan genes in a genome.

ORFans are defined as the genes sharing no similarity with genes or coding sequence domains in other evolutionary lineages [12, 13]. They have no recognizable homologs in distantly related species. This definition is conceptually simple, but operationally complex. The identification of ORFans depends both on the detection method and on the reference set of genomes because this defines the evolutionary lineage to be investigated. Albà and Castresana [16] questioned whether BLAST was a suitable procedure to detect all of the true homologues and they concluded that BLAST was a proper algorithm to identify the majority of remote homologues (if they existed). Tautz and Domazet-Lošo developed a general framework, the so-called “phylostratigraphy” [17], which consists of statistical evaluation of macro-evolutionary trends [17–19]. Phylostratigraphy is being applied for systematic identification of all orphan genes within the evolutionary lineages that have led to a particular extant genome [18–23].

Lipman et al. [1] studied the length distributions of the Having Homologs Proteins (HHP) and Non-Conserved Proteins (ORFans in our nomenclature) sets for the bacterium *Escherichia coli*, the archaeon *Archaeoglobus fulgidus*, and three eukaryotes. Regarding the two prokaryotes, the group made the following observations:

i. HHPs are, on average, longer than ORFans.
ii. The length distribution of ORFans in a genome has a relatively narrow peak, whereas the HHPs are spread over a wider range of values.

Limpan’s observations has been made in 2002, before the research community realized that short ORFs are in fact real and code for functional proteins or small RNA. The conclusions, therefore, may have been biased by automated procedures for genome annotation discarding proteins shorter than 50 aa, or even 100 aa [24]. Therefore, many short proteins could have been mistakingly labeled as ORFans, since their orthologs in other species were not identified. Small peptide-coding open reading frame sequences were too short for computational analysis, and hence were frequently mis-annotated and under-represented proteomics datasets in spite of their important roles in cell biology [25]. Recent studies demonstrated that small proteins, most of which act at the membrane, can no longer be ignored [24–28]. This sequencing bias, however, cannot explain the difference between *Mycoplasmataceae* and other prokaryotic families.

Lipman et al. [1] proposed that there is a significant evolutionary trend favoring shorter proteins in the absence of other, more specific functional constraints. However, so far, research in this area is limited in the scope of organisms. Here, we have tested the above-mentioned observations by Lipman et al. [1] on a comprehensive set of all sequenced and annotated bacterial genomes. We performed comparisons of length distributions of HHP and ORFans in all annotated genomes and confirmed, to a large extent, the conclusions of Lipman et al. [1]. Below, we described and discussed the few remarkable exceptions to the general rules.

## Results and discussion

The majority of species, forming exceptions to the Lipman’s rule [1], belong to the *Mycoplasmataceae* family. *Mycoplasmataceae* lack the cell wall, featuring some of the smallest genomes known and are “metabolically challenged”, i.e. missing some essential pathways of free-living organisms [29–33]. Many *Mycoplasmataceae* species are pathogenic in humans and animals.

## HHPs and ORFans lengths

**Fig. 1:**
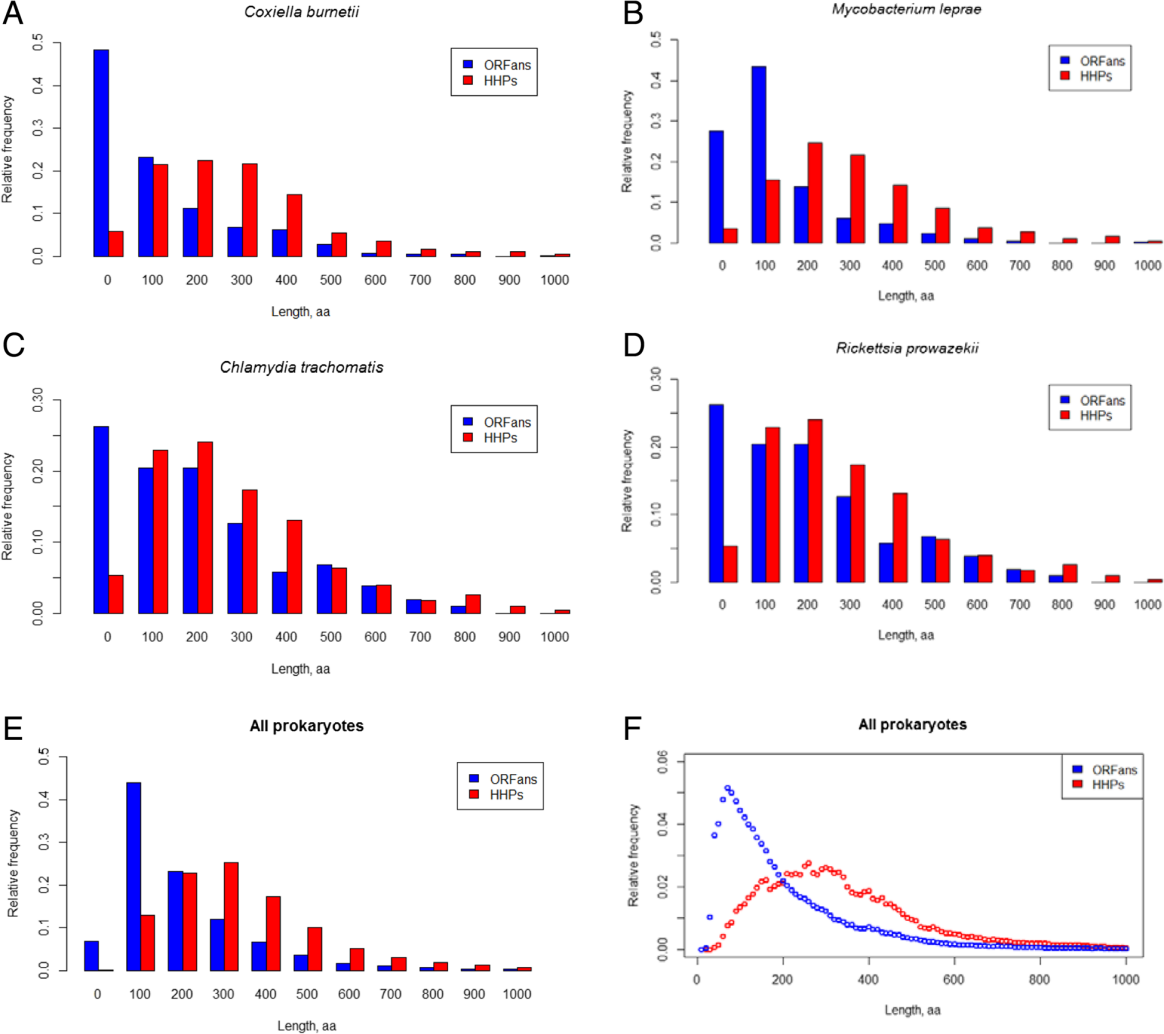
Histograms of protein lengths of *Coxiella burnetii* (**a**), *Mycobacterium leprae* (**b**), *Chlamydia trachomatis* (**c**), *Rickettsia prowazekii* (**d**) and all other prokaryotes (**e**) and (**f**) tested in this study. The X axis corresponds to the protein length intervals (0 -- (0100], 100-- (100,200], etc.), while the Y axis show the relative frequency of ORFans and HHPs among the genomes. Bar plot and the relative frequency plot with a smaller bin size for all prokaryotes are presented on Panels (**e**) and (**f**), respectively

We have selected four genomes out of the currently sequenced and annotated 1484 bacterial genomes to illustrate typical protein lengths distributions for HHPs and ORFans, (Fig. 1, Panels a–d). The ORFans’ length distributions are relatively narrow, in contrast to the HHPs, which lengths spread over a wider range of values. ORFans are obviously shorter than HHPs in all four species (Fig. 1, Panels a–d). Note that the distributions of protein lengths in the four selected bacteria are similar to the global distribution presented in Fig. 1 (Panels e–f).

**Fig. 2:**
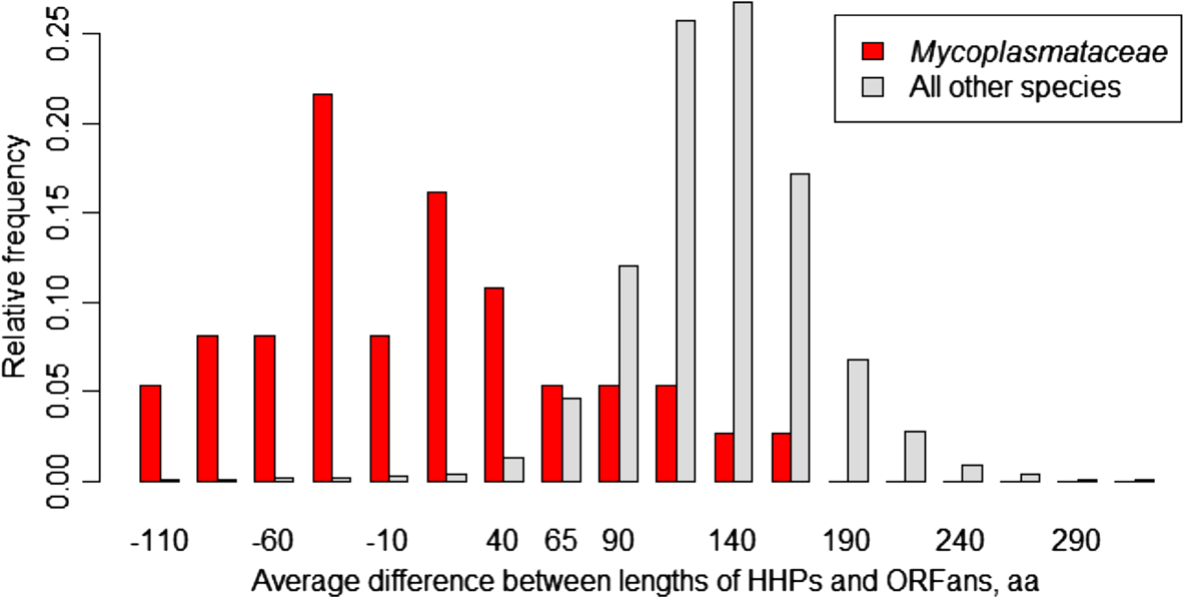
The difference between mean lengths of HHPs and ORFans for 1484 prokaryotic genomes. The X axis corresponds to the difference between mean length of HHPs and ORFans, and the Y axis shows the relative frequency of genomes with the given length difference. For all prokaryotic genomes, HHPs are longer than ORFans by, on average, 144 amino acids. For the *Mycoplasmataceae* genomes, the average difference is only 14 amino acids, while 17 out of 37 *Mycoplasmataceae* genomes have ORFans that are, on average, longer than HHPs

Based on the data from two genomes, Lipman et al. [1] suggested that HHPs are, on average, longer than the ORFan proteins, in general. In order to test this statement, we have calculated distributions of protein lengths for all COG-annotated genomes, and built a histogram of differences between the means of HHPs and ORFans, which happened to be approximately bell-shaped (Fig. 2). On average, HHPs are longer than ORFans by approximately 150 amino acids. However, the distribution has a heavy left tail containing genomes with the ORFan’s mean length equal to or exceeding the HHP’s mean length (Fig. 2). See Additional file 1 for additional discussion and the complete dataset.

In order to investigate this effect, we sorted the genomes according to the difference between the mean lengths of HHPs and ORFans (Table 1). Most “atypical genomes” with longer ORFans belong to the species from the *Mycoplasmataceae* family (*Mycoplasma* and *Ureaplasma* genera) and some to the *Anaplasmataceae* family (*Anaplasma* and *Ehrlichia* genera). There are also solitary representatives of other lineages: *Chlorobium chlorochromatii, Lawsonia intracellularis, Burkholderia pseudomallei, Rhodobacter sphaeroides,* and *Methanobrevibacter ruminantium.* Appearance of these solitary representatives may be explained by different factors, including random ones. Anyway, only two taxons (the *Mycoplasmataceae* and *Anaplasmataceae* families) massively present among atypical genomes, while only the *Mycoplasmataceae* family contains 32 fully sequenced and annotated genomes with atypical ORFans, which is sufficient for statistical analysis (see Table 2). Therefore, we restricted our analysis to the *Mycoplasma* and *Ureaplasma* genera.

**Table 1:** List of atypical genomes showing HHPs’ average length, number of HHPs, ORFans' length, and the difference between the average length of HHP and ORFans for a given genome

	Species	Average length of HHPs, aa	Number of HHPs	Average length of ORFans, aa	Number ORFans	Difference between the average length of HHP and ORFans, aa
1.	*Ureaplasma urealyticum serovar 10 ATCC 33699 uid59011*	366	416	472	230	−106
2.	*Mycoplasma genitalium G37 uid57707*	350	384	450	91	−100
3.	*Mycoplasma hyopneumoniae 7448 uid58039*	375	443	449	214	−74
4.	*Anaplasma centrale Israel uid42155*	349	691	417	232	−68
5.	*Mycoplasma gallisepticum R low uid57993*	368	489	429	274	−61
6.	*Ureaplasma parvum serovar 3 ATCC 27815 uid58887*	359	413	410	196	−51
7.	*Chlorobium chlorochromatii CaD3 uid58375*	367	1564	417	435	−50
8.	*Anaplasma marginale Florida uid58577*	353	699	395	241	−42
9.	*Mycoplasma mobile 163 K uid58077*	358	450	400	183	−42
10.	*Mycoplasma hyorhinis HUB 1 uid51695*	352	464	387	194	−35
11.	*Mycoplasma hyopneumoniae 232 uid58205*	375	437	410	254	−34
12.	*Ureaplasma parvum serovar 3 ATCC 700970 uid57711*	363	441	394	173	−30
13.	*Anaplasma marginale Maries uid57629*	352	699	382	249	−30
14.	*Mycoplasma conjunctivae uid59325*	360	420	387	272	−27
15.	*Mycoplasma crocodyli MP145 uid47087*	363	490	387	199	−24
16.	*Mycoplasma hyopneumoniae J uid58059*	391	471	413	186	−23
17.	*Mycoplasma hominis ATCC 23114 uid41875*	369	378	383	145	−14
18.	*Lawsonia intracellularis PHE MN1 00 uid61575*	492	51	500	53	−8
19.	*Mycoplasma penetrans HF 2 uid57729*	384	658	390	379	−5
20.	*Burkholderia pseudomallei 1710b uid58391*	377	2835	374	898	2
21.	*Mycoplasma putrefaciens KS1 uid72481*	358	474	351	176	7
22.	*Mycoplasma agalactiae uid46679*	366	522	354	291	11
23.	*Rhodobacter sphaeroides 2 4 1 uid57653*	332	82	318	21	13
24.	*Methanobrevibacter ruminantium M1 uid45857*	348	1513	335	704	14
25.	*Nanoarchaeum equitans Kin4 M uid58009*	286	356	269	184	16
26.	*Mycoplasma synoviae 53 uid58061*	363	479	345	180	17
27.	*Mycoplasma mycoides capri LC 95010 uid66189*	384	619	361	303	23
28.	*Mycoplasma agalactiae PG2 uid61619*	353	475	329	267	25
29.	*Mycoplasma bovis PG45 uid60859*	371	526	343	239	28
30.	*Ehrlichia canis Jake uid58071*	348	678	320	247	28
31.	*Mycoplasma pulmonis UAB CTIP uid61569*	379	560	350	222	30
32.	*Mycoplasma pneumoniae M129 uid57709*	367	445	334	203	32

**Table 2:** Number of sequenced and annotated genomes for the selected set of bacterial species

Genus	Total number sequenced genomes	Number of genomes with assigned COG
*Anaplasma*	9	4
*Ehrlichia*	6	5
*Neorickettsia*	2	2
*Wolbachia*	7	4
*Mycoplasma*	68	29
*Ureaplasma*	3	3
*Mesoplasma*	2	1
*Spiroplasma*	6	0
*Acholeplasma*	3	1
*Candidatus Phytoplasma*	3	2
*Anaeroplasma*	0	0
*Asteroplasma*	0	0
*Entomoplasma*	0	0

## Variability of protein lengths

The *Mycoplasmataceae* genomes challenge the second conclusion of Lipman et al. [1] about the length distributions of ORFans and they have a relatively narrow peak, whereas those of the HHP are spread over a wider range of values. The histogram of differences between HHPs and ORFans in these atypical genomes is shown in Fig. 2 (red bars). We calculated the Coefficient of Variation ($CV=\frac{sd(Y)}{\overline{Y}}$, where Y is a set of protein lengths); average difference between CV for ORFans and HHPs in “atypical” genomes was 0.31. We also computed variances of lengths for ORFans and HHPs separately and conducted the F-test, resulting in *p*-values <10^−64^ for all tested pairs. Therefore, the ORFan proteins of these genomes are more variable in length than the HHPs.

## Selection of a statistic for identification of atypical genomes

**Fig. 3:**
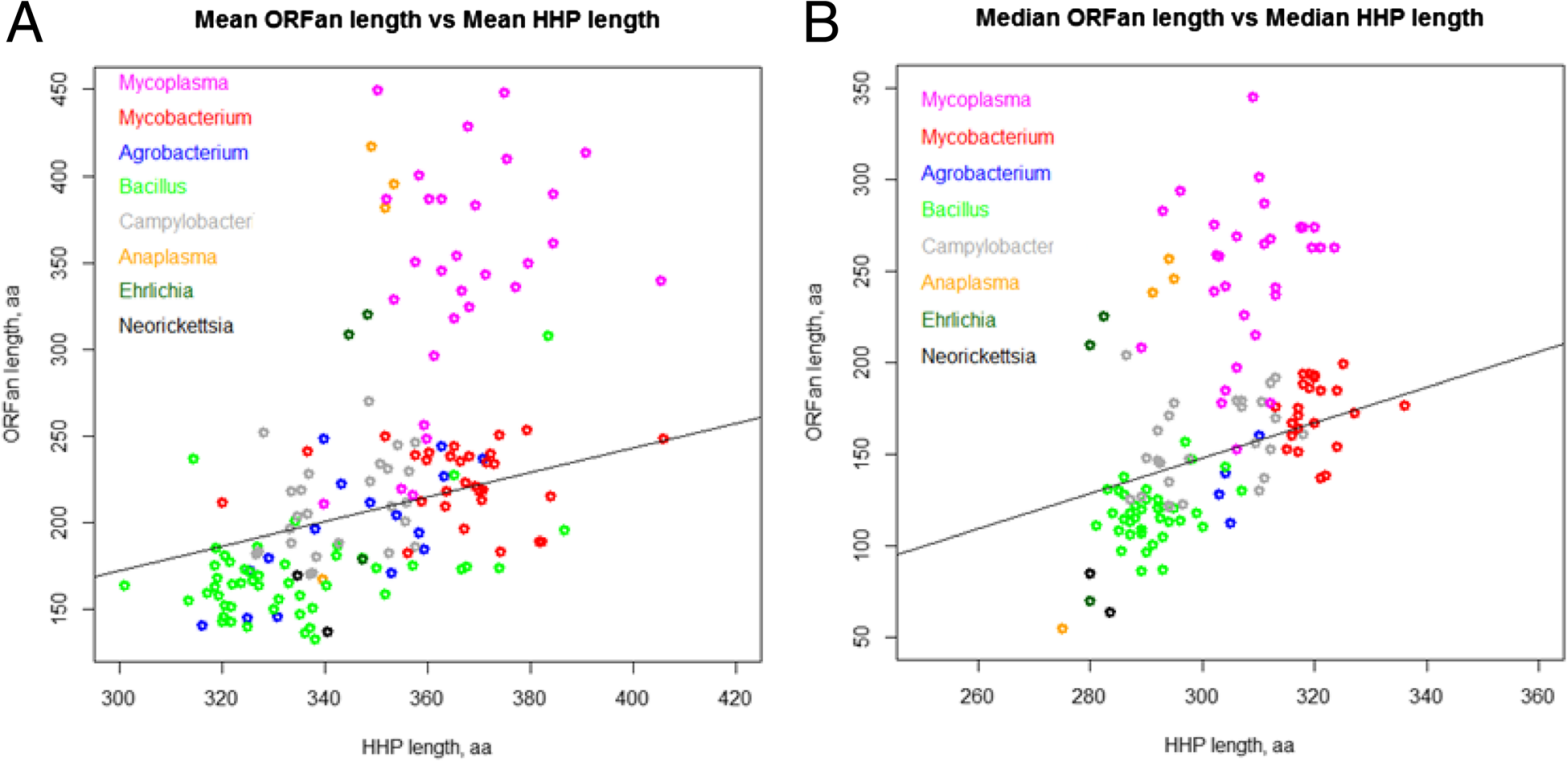
Mean (**a**) and Median (**b**) ORFans’ length vs. average HHP length for selected eight groups of prokaryotes. Each point represents a genome. Family *Mycoplasmataceae* (pink), family *Mycobacteriaceae* (red), genus *Agrobacterium* (blue), genus *Bacillus* (green), genus *Anaplasma* (orange), genus *Ehrlichia* (dark green), genus *Neorickettsia* (black) and genus *Campylobacter* (grey) are shown. The regression line shows the relationships between the mean HHP length and the mean ORFan length across 1484 annotated prokaryotic genomes

We tested the relationships between the mean HHP length and the mean ORFan length on eight groups of prokaryotes: two families of *Mycoplasmataceae* and *Mycobacteriaceae*, six genera *Agrobacterium, Bacillus, Anaplasma, Ehrlichia, Neorickettsia* and *Campylobacter* (Fig. 3, Panel a). *Mycoplasmataceae* genomes form a clearly distinct group of atypical genomes. As shown below, there are a small number of unusually long ORFan proteins in *Mycoplasmataceae*, the outliers that may skew the distribution. Therefore, considering only the mean gene lengths distribution may be insufficient; the median value is probably a more appropriate measure (Fig. 3, Panel b). However, again, the *Mycoplasmataceae* represent a group of atypical genomes. Therefore, poorly predicted outliers in the *Mycoplasmataceae* genomes cannot exclusively explain the effect.

It is worth mentioning that outside of the *Mycoplasma* and *Ureaplasma* genera, there is one currently sequenced bacterial genome with the ORFans’ mean length larger than the HHP’s. In *Candidatus Blochmannia floridanus*, the former value is twice as large as the mean length of HHPs, due to the only unusually large ORFan protein of 680 amino acids (while the average HHP length for *Candidatus Blochmannia floridanus* is 334 aa, with the median length of 294 amino acids (aa), and the longest protein is 1420 aa).

**Fig. 4:**
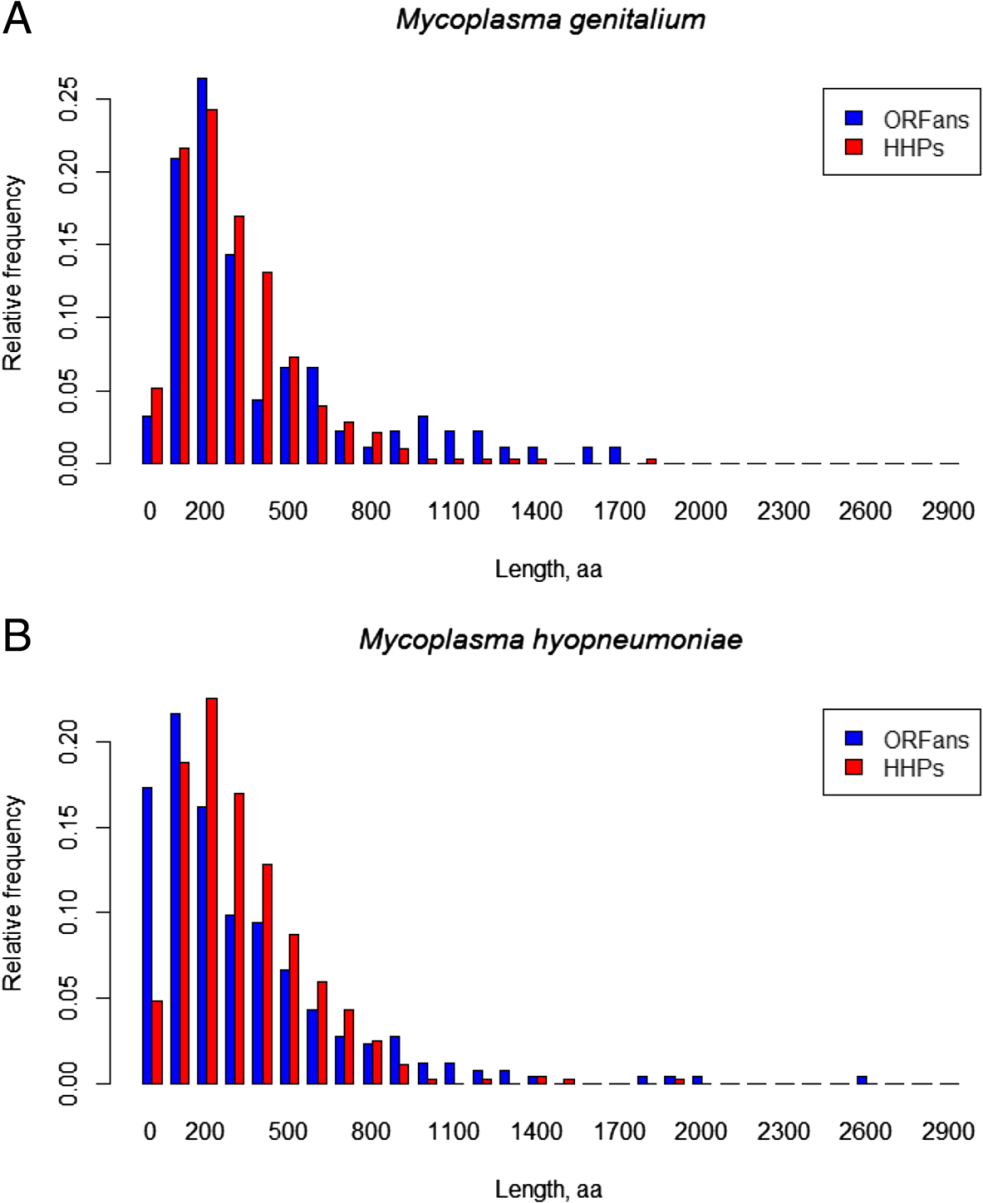
Histograms of protein lengths of (**a**) *M. genitalium* (*M. genitalium* G37 uid57707, NC_000908) and (**b**) *M. hyopneumonia* (*M. hyopneumoniae* 232 uid58205, NC_006360) X axis labels correspond to the following protein length intervals 0 -- (0100], 100-- (100,200], etc. Y axis shows a relative frequency of the protein with a given length in a genome

Typical distributions of the protein lengths of HHPs and ORFans in *Mycoplasmataceae* are illustrated by two genomes (*M. genitalium* and *M. hyopneumonia*), selected out of 68 sequenced genomes of *Mycoplasmataceae* (Fig. 4) These ORFans’ distributions are rather different from the ones for four bacteria shown above (Fig. 1, Panels a–d). *Mycoplasma* protein length distributions have two properties that distinguish them from other organisms:

i. Existence/presence of few very short ORFans in Mycoplasma’ genomes. On average, *Mycoplasma* species contain 44 ± 12 short (length <100 aa) proteins per genome as compared to 145 ± 7 short proteins per genome in all other sequenced prokaryotes.
ii. Comparatively, many ORFans longer than 800 aa (on average, *Mycoplasma* species contain 17 ± 4 long (≥800 aa) proteins per genome as compared to 11 ± 1 long proteins per genome in all other sequenced prokaryotes). Moreover, there are several extremely long ORFans. On average*, Mycoplasma* species contain 10 ± 1 very long (≥1000 aa) proteins per genome as compared to 6 ± 1 very long proteins per genome for all other sequenced prokaryotes.

## Functional annotation of ORFans

We selected 9350 ORFans out of 32 species from the *Mycoplasmataceae*, which found the best hits in other prokaryotic genomes, and stratified them by functional annotation in the COG database. 54 % of ORFans were mapped to a “hypothetical protein” category: 6 % are ‘lipoproteins”, further, 2 % are “membrane lipoproteins”, 3 % are “surface protein 26-residue repeat-containing proteins”, and the rest is mapped to lesser-abundant categories. A protein is called “hypothetical” if its existence has been predicted *in silico*, but the function is not experimentally validated. Despite that *Mycoplasmataceae* cells are wall-less with no periplasmic space, they effectively anchor and expose surface antigens using acylated proteins with long-chain fatty acids [34–36]. Lipoproteins are abundant in mycoplasmal membranes and are considered to be key elements for diversification of the antigenic character of the mycoplasmal cell surface [34, 37].

For the long proteins (≥1000 aa) we are especially interested in, we compared the functional annotations between HHPs and ORFans. These two groups were most different than those in the “hypothetical protein” category (*p*-value = 4.00195E-21), overrepresented in ORFans, followed by “efflux ABC transporter, permease protein”, and also over-represented in the long ORFans of *Mycoplasmataceae* (*p*-value =4.18953E-06). Тhe best BLAST hits of *Mycoplasma*’s “efflux ABC transporter, permease proteins” were to the ABC transporter proteins from two related species, *Ureaplasma parvum* and *Ureaplasma urealyticum.* Moreover, the multiple protein alignment of the CLUSTALW (see Additional file 1) shows a high degree of conservation among the “efflux ABC transporter, permease proteins” across all genomes of *Mycoplasmataceae*.

## Are the observed peculiarities features of *mycoplasmataceae* family or the entire class of *mollicutes*?

In order to investigate whether long ORFans are a specific feature of the *Mycoplasmacaea* or not*,* we analyzed ORFans’ sizes in several species from the same *Mollicutes* class, including *Acholeplasma* and *Candidatus Phytoplasma*. In *Candidatus Phytoplasma australiense* and *Acholeplasma laidlawii*, ORFans are 1.5–2 times shorter than HHPs. Therefore, we concluded that these features are not universal for *Mollicutes*.

We have also analyzed the genomes of *Anaplasma* that belong to the family *Ehrlichiaceae* in the order of *Rickettsiales*. The genus, *Anaplasma,* includes obligatory parasitic intracellular bacteria, residing in the vacuoles in eukaryotic host cells and lacking stained cytoplasm. Out of four representatives of the *Anaplasma* genus with sequenced genomes, *A. marginale* (two strains) and *A. centrale* cause anaplasmosis in cattle, while *A. phagocytophilum* causes anaplasmosis in dogs, cats and horses. ORFans of *A. marginale* and *A. centrale* are, on average, 47 aa longer than HHPs. ORFans of *A. phagocytophilum* are, on average, 172 aa shorter than the HHPs. There are three times more HHPs than ORFans in *A. marginale* and *A. centrale,* while in *A. phagocytophilum* this ratio is equal to 1.3, while the total number of HHPs is approximately the same. Therefore, the effect can be attributed to either annotation artifact or to host specificity.

On average, in *A. centrale*, the ORFan proteins are 68 aa longer than of HHP proteins, and in *A. marginale* ORFans are 42 aa longer than HHPs. However, the median protein lengths of ORFans are 40 and 50 aa shorter than HHPs in both considered *Anaplasma* genomes, correspondingly (see Fig. 3). This discrepancy is due to several unusually long ORFan proteins with hypothetical function that skew the mean length up. Moreover, the ORFans feature shorter mean and median lengths than HHPs in all tested *Ehrlichia* and *Neorickettsia* species (*Ehrlichia canis, Ehrlichia ruminantium, Ehrlichia chaffeensis, Neorickettsia risticii, Neorickettsia sennetsu*). These bacteria (together with *Anaplasma* species) belong to the order *Rickettsiales*. Two strains of *Ehrlichia ruminantium Welgevonden* were excluded due to an inconsistency of annotations between them. Based on the data obtained, we concluded that the phenomenon of extremely long ORFans is specific for the family of *Mycoplasmataceae.*

## Driving forces behind the long ORFans

Why do the *Mycoplasmataceae* have ORFans as long as HHPs with the distribution of ORFans’ lengths very similar to HHPs? *Mycoplasmataceae* are a heterogeneous group of the cell-wall-less, the smallest and the simplest self-replicating prokaryotes. They have a reduced coding capacity and have lost many metabolic pathways, as a result of a parasitic lifestyle [38, 39]. These organisms are characterized by a lack of a cell wall, a small genome size, a low G + C content (23 to 40 %) and an atypical genetic code usage (UGA encodes tryptophan instead of a canonical opal stop codon) [40]. In addition, *Mycoplasmataceae* genomes lack 5′ UTRs in mRNAs, as established by Nakagawa et al. [41]. This phenomenon is highly unusual in bacteria. Below we propose and discuss several reasons that might explain the presence of long ORFans in *Mycoplasmataceae*.

### Small genome size

Prokaryotic genomes range from 10Kbp (*Bacteroides uniformis*, associated with the degradation of the isoflavone genistein in human feces) to 39 Mbp (*Vibrio parahaemolyticus*, causing acute gastroenteritis in humans), with the mean length of 3.5 Mbp and the median of 3.0 Mbp [42]. *Mycoplasmataceae*, indeed, tend to have small genomes (mean/median lengths are 0.9 Mbp, minimum is 0.58 Mbp, and the maximum is 1.4 Mbp). However, there are many bacteria with smaller genomes, including such “dwarfs” as *Candidatus Tremblaya princeps* and *Candidatus Hodgkinia cicadicola* (0.14 Mbp each), and *Candidatus Carsonella ruddii* (0.17 Mbp). The “genomic dwarfism” per se is not associated with unusual ORFans. Among the 324 annotated “genomic dwarfs” with genome sizes below 2 Mbp, only *Ureaplasma, Anaplasma* and *Mycoplasma* genomes feature the average ORFan length to be over 95 % of the average HHP length. In all other species (except one), the ratio of ORFan to HHP length ranges from 30 to 90 %. The exception is a tiny (400 nm in diameter) marine Archaeon, *Nanoarchaeum equitans* with the average ORFans’ length of 94 % of the HHPs’ length. N*anoarchaeum* is a remarkable organism; it is an obligate symbiont on the archaeon *Ignicoccu*s, which cannot synthesize lipids, amino acids, nucleotides, or cofactors [43].

Neither the β-proteobacterium *Candidatus Tremblaya princeps* (endosymbiotic bacteria living inside the citrus pest mealybug *Planococcus citri*), *Candidatus Carsonella ruddii* (endosymbiont present sap-feeding insects psyllids), nor the *Candidatus Hodgkinia cicadicola* (α-proteobacterial symbiont of cicadas) features an unusually long length of ORFans. For all three species, the mean ORFan length is approximately 40 % of the HHP length. Therefore, we conclude that the small genome size alone cannot explain the presence of long ORFans in *Mycoplasmataceae*.

### Low GC content and unusual base composition in a reduced bacterial genome

We analyzed 300 genomes with the lowest GC content (ranging from 14 to 36 %), including three species of *Ureaplasma* and 77 species of the *Mycoplasma*. Overall, there is only а weak positive correlation (Pearson’s correlation coefficient *ρ* = 0.13) between the GC-content and the ORFan to HHP length ratio, and plenty of examples of GC-poor genomes with low ORFan to HHP length ratio. The GC-poor species features an average ORFans to the average HHPs ratio of 60 %, ranging from 20 to 106 %. Among ten most GC-poor genomes, the ORFan to HHP length ratio varies between 30 and 76 %.

The GC-poor *Ureaplasma* and *Mycoplasma* species have average ORFans to HHPs ratio of 98 %, the lowest being 61 % and the highest 130 %. Interestingly, among the 300 GC-poor species the upper tail of the high ORFan to HHP length ratio is occupied by *Ehrlichia ruminantium, Ehrlichia canis, Methanobrevibacter ruminantium,* and *Nanoarchaeum equitans. E. ruminantium* and *E. canis* belong to the *Anaplasmataceae* family; they are obligatory intracellular pathogens transmitted by ticks. According to Mavromatis et al. [44], *E. canis* genome contains a large number of proteins with transmembrane helices and/or signal sequences and a unique serine-threonine bias prominent in proteins associated with pathogen-host interactions.

The GC_3_ is defined as a fraction of guanines and cytosines in the third codon position [45]. The importance of the variability in the genomic GC and the genic GC_3_ content for stress adaptation has been established by multiple authors for a number of prokaryotic and eukaryotic organisms [46–50]. The mechanisms behind GC-content differences in bacterial genomes are unclear, although variability in the replication and/or repair pathways is suggested as a hypotheses [51–53]. One mechanistic clue is the positive correlation between the genome size and GC content (smaller genomes tend to have lower GC-content). This tendency is particularly pronounced for obligate intracellular parasites. Two (not necessarily mutually exclusive) hypotheses have been forwarded to explain this base composition bias in the genomes of intracellular organisms. The first is an adaptive hypothesis, based on selection for energy constraints [54]. It says that the low GC content helps the intracellular parasites to compete with the host pathways, for the limited metabolic resources in the cytoplasm. The second hypothesis relates to the mutational pressure, which results from the limited DNA repair systems in the bacterial parasites [55]. Small intracellular bacteria often lose non-essential repair genes, and, therefore, are expected to be deficient in their ability to repair damage caused by spontaneous chemical changes. This is particularly expected for the endosymbionts, in which the genetic drift plays a major role in sequence evolution [55].

**Fig. 5:**
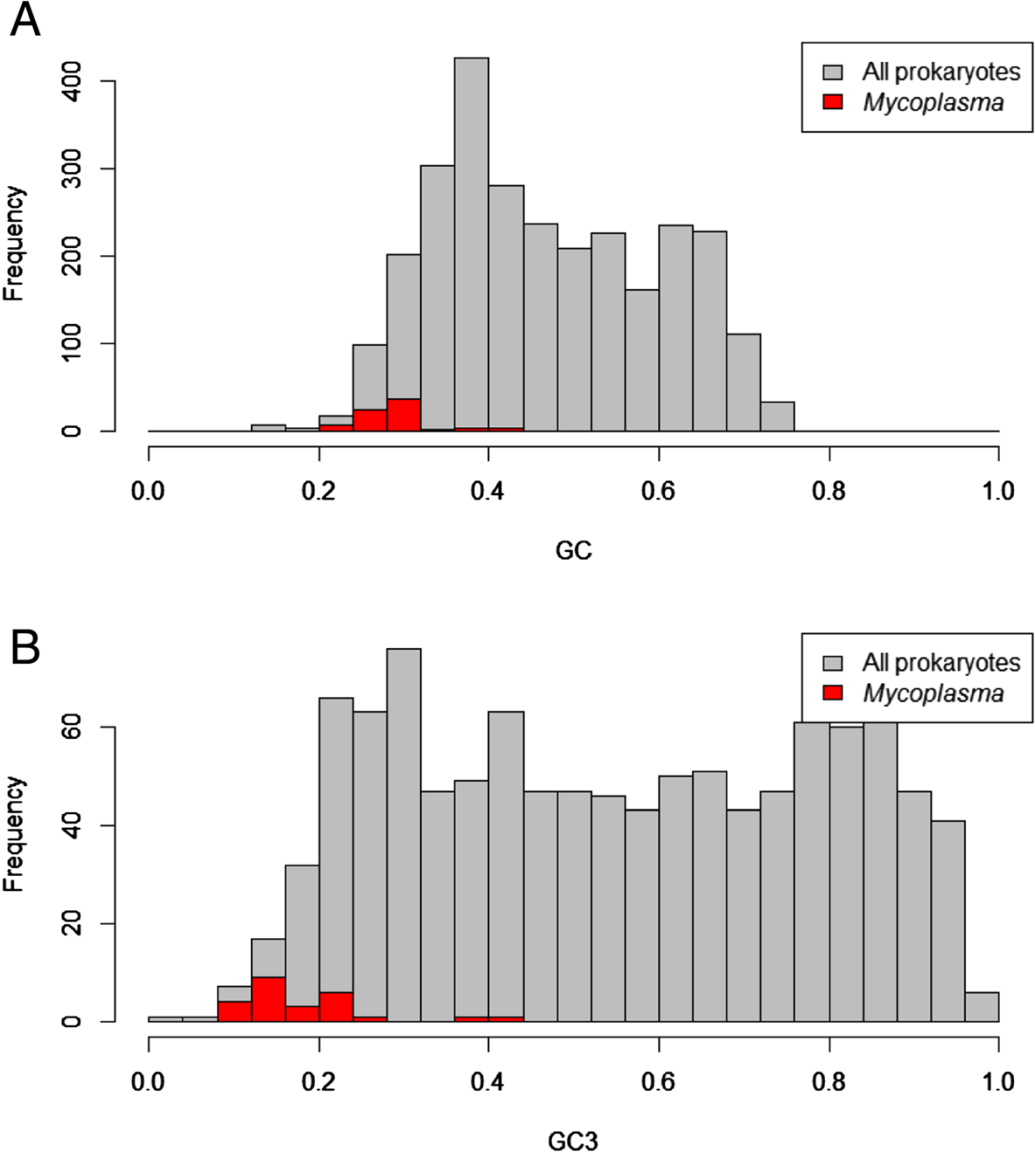
Genomic GC content (Panel **a**) and genic GC_3_ content (Panel **b**) in annotated species of *Mycoplasmataceae*. Grey histograms correspond to all prokaryotes while red histograms correspond to selected *Mycoplasma* species. Horizontal axis shows GC (**a**) and GC_3_ content, and vertical axis shows the number of prokaryotic genomes with the given content

Thus, the *Mycoplasma,* and the *Ureaplasmae* are the GC and GC_3_ – poor (Fig. 5, Additional file 2: Table S1). Why is the GC-poverty so important? According to the “codon capture model”, in the GC–poor environment, the replication mutational bias towards the AT causes the stop codon of the TGA to change to the stop codon of the TAA, without affecting the protein length [56, 57]. The subsequent appearance of the TGA codon through a point mutation leaves it free to encode for an amino acid (Trp). This brings us to our next point of discussion.

In the *Mycoplasmataceae,* the ORFans have a 3 % lower GC content than the HHPs do. This is close to the average difference in the GC content for ORFan genes in all prokaryotic species (−3.9 %, with the range from −25 to 25 %). Some species with the lowest GC-content in ORFans are: *Dickeya dadantii, Citrobacter rodentium, Pectobacterium wasabiae, Chromobacterium violaceum, Alicyclobacillus acidocaldarius, Neisseria meningitidis,* and *Shigella sonnei.* The species with the highest GC content in ORFans include: *Methylobacterium chloromethanicum CM4, Escherichia coli SE11, Lactobacillus plantarum, Spirosoma linguale* and *Bifidobacterium longum infantis.*

The variability of the GC_3_ content in bacteria appears to be an instrument of environmental adaptation, allowing to keep the protein sequence unchanged. According to Mann and Chen [58], in nutrient-limited and nutrient poor environments, the smaller genome size and the lower GC content help to conserve replication expense. Generally, species with many GC_3_-rich genes have ORFans with lower GC_3_ contents, and species with many GC_3_-poor genes (average GC_3_ < 0.3) have ORFans with the same or higher GC_3_ contents as HHPs do. We observed that, on average, prokaryotic ORFans have a 12.5 % lower GC_3_ content as compared to HHPs of the same organism. Some species (such as *Burkholderia pseudomallei, Burkholderia mallei, Thermobispora bispora, Burkholderia pseudomallei, Chromobacterium violaceum, Rhodobacter sphaeroides,* and *Kineococcus radiotolerans*) feature a two-fold decrease in GC_3_ content of ORFans, compared to the HHPs*.* ORFans of some other species have a higher GC_3_ content than HHPs (20 % increase or more). These include: *Methylobacterium chloromethanicum, Pelagibacterium halotolerans, Escherichia coli SE11, Lactobacillus plantarum,* and *Thermofilum pendens.* Curiously, ORFans of the *Mycoplasma* and the *Ureaplasma* have the same GC_3_ content as their HHPs (around 20 %). It appears that, since the genes of *Mycoplasma* and *Ureaplasma* already have a low GC_3_ content, there simply is no more room to decrease it further for the ORFans.

Based on these findings, we conclude that the GC-content of genes and genome cannot be a sole factor responsible for the existence of long ORFans in a *Mycoplasmataceae*.

### UGA StopRTrp recoding

Almost all bacterial and archaeal species have three stop codons: TAA, TGA and TAG. However, there are 77 exceptions to this rule among the currently completely sequenced 2723 prokaryotic genomes (note that only 1484 of them are COG-annotated and, therefore, were used in our study). Seventy-three species out of 77 belong to the genera *Mycoplasma, Spiroplasma*, and *Ureaplasma*; all of them are small bacteria of the class *Mollicutes*. In addition, in several mitochondrial lineages, the UGA StopRTrp recoding is also associated with both genome reduction and low GC content [59–61]. For example, *Candidatus Hodgkinia cicadicola*, mentioned above because of its “dwarf genome”, it also features the coding reassignment of the UGA Stop → Trp [62]. Moreover, two groups of currently uncultivable bacteria, found in marine and fresh-water environments and in the intestines and oral cavities of mammals, use UGA as an additional glycine codon instead of a signal for translation termination [63]. Under the “codon capture” model, a codon falls to low frequency and is then free to be reassigned without major fitness repercussions. Applying this model to the UGA StopRTrp recoding, mutational bias towards the AT causes each UGA to mutate to the synonym UAA without affecting its protein length [56, 57]. When the UGA codon subsequently reappears through a mutation, it is then free to encode for an amino acid [56, 57]. While some have argued that codon capture is insufficient to explain many recoding events [2, 59, 60], the fact that all known UGA StopRTrp recoding has taken place in low GC genomes [59, 64] makes the argument attractive for this recoding. It was suggested [56] that the recoding was driven by the loss of translational release factor RF2, which recognizes the TGA stop codon. Notably, despite the fact that the *Candidatus Hodgkinia cicadicola* uses the UGA StopRTrp recoding, it has a perfectly normal difference of distributions between ORFans and HHPs [5]. According to Ivanova et al. [65], the TGA reassignment is likely to be limited to the *Mollucites* and *Candidatus Hodgkinia cicadicola,* and it occurred as a single event after the last common ancestor separated from the *Peregrine*s group.

We have also examined other members of *Mollicutes* and found numerous examples when the distribution of differences between ORFans and HHPs was normal. Since 73 out of 77 species with TGA reassignment are *Mycoplasma* and *Ureaplasma* species, there is not enough statistical power/data to conclude whether recoding of UGA StopRTrp is the main cause of long ORFans.

### Lack of a cell wall and parasitic lifestyle

Several bacterial species have wall-less cells (L-forms), as a response to extreme nutritional conditions [66]; L-forms might have played a role in the evolution of the bacterial species, with respect to the emergence of the *Mycoplasma* [67]. In order to compensate for the lack of the cell wall, the *Mycoplasma* developed extremely tough membranes that are capable of contending with the host cell factors. Lipoproteins are abundant in mycoplasmal membranes [34, 37]. They modulate the host’s immune system [68], therefore playing an important role in the infection propagation. The ability of lipoproteins to undergo frequent size or phase variation is considered to be an adaptation to different conditions, including the host’s immune response [68, 69]. Some of the largest gene families in the *Mollicute* genomes encode ABC transporters, lipoproteins, adhesins and other secreted virulence factors [36]. This may be due to the absence of a cell wall and a periplasmic space in the *Mollicute*, attributable to their parasitic lifestyle in a wide range of hosts. We identified many of the *Mollicutes* ORFans as hypothetical proteins and lipoproteins in the COG functional classification. Moreover, with hypothetical proteins and an efflux ABC transporter, the permease proteins were predominant among the longest proteins (≥1000 aa). Hypothetical proteins constitute a large group proteins in the *Mollicutes* [70–72]. Lipoproteins, especially membrane exposed ones, are abundant in the *Mollicutes*, in sharp contrast to other bacteria, which only have a limited number of lipoproteins in the membranes [36]. In general, lipoproteins carry out numerous important functions, including protection against osmotic and mechanical stress and interactions with the host [36]. However, most *Mollicute* lipoproteins currently lack the exact functions and their host protein interaction partners are unknown. Depending on the species, lipoproteins are encoded by single or multiple genes (multi-gene families) and some of them are members of the paralogous families, such as the P35 lipoprotein of *M. penetrans* [73]. Some lipoproteins are species-specific, while others have homologs that are among different species. In particular, they are associated with or share a sequence similarity with the ABC transporter genes, suggesting that they may play a role in the transport of nutrients into the cell [74]. It is a well established fact that prokaryotic ABC transporters translocate different compounds across cellular membranes in an ATP coupled process (a crucial function for obligate parasites like *Mollicutes*). They also carry out a remarkable diversity of other functions, some of which are essential for pathogenicity [75].

The accessory genes or ORFans are usually important sources of genetic variability in bacterial populations, which are thought to play a role in niche adaptation, host specificity, virulence, or antibiotic resistance. Most of the identified *Mycoplasmataceae* ORFans are surface exposed proteins, suggesting that they may play a role in shielding the wall-less mycoplasma cell membrane from a host defense. Interestingly, the long variable lipoproteins (Vlp) of *Mycoplasma hyorhinis*, such as variants expressing longer versions of VlpA, VlpB, or VlpC are completely resistant to growth inhibition by host antibodies, unlike their shorter allelic versions [76, 77]. The same effect was observed for variable surface antigens (Vsa) of *Mycoplasma pulmonis,* in which the long Vsa variants are highly resistant to complement lysis while the shorter variants are susceptible [78].

From this discussion, it is not surprising that lipoproteins in the *Mycoplasmataceae* have many unusual properties, including the gene lengths distribution. Being unique, these proteins cannot be assigned to any COG, which results in classifying them as ORFans. Certainly, more studies should be carried out to clarify why the *Mycoplasmataceae* contain long ORFans in comparison to other bacteria.

## Conclusions

We have compared the lengths’ distributions of “having homologs proteins” (HHPs) and “non-having homologs proteins” (orphans or ORFans) in all currently annotated completely sequenced prokaryotic genomes.

In general, we confirmed that the findings of Lipman et al. [1] are established on a limited set of genomes that: (1) HHPs are, on average, longer than ORFans; (2) In a given genome, the length distribution of ORFans has a relatively narrow peak, whereas the HHPs are spread over a wider range of values. We have shown that about thirty genomes do not obey the “Lipman rules”. In particular, all genomes of the *Mycoplasma* and *Ureaplasma* have atypical ORFan distributions, with the mean lengths of ORFan’s being larger than that of the mean lengths of HHPs. We established that these differences cannot be explained by the “usual suspects” hypotheses of small genome size and a low GC content of the *Mycoplasmataceae. Mycoplasmataceae* is a heterogeneous group of the smallest and simplest self-replicating prokaryotes with limited metabolic capabilities, which parasitize a wide range of hosts [38, 39]. These organisms are characterized by a lack of a cell wall, they have small genome sizes, they have a low GC content (23 to 40 %) of the genome, and the usage of different genetic code (usage UGA as a tryptophan codon instead of the universal opal stop codon) [34].

We propose that the atypical features of the *Mycoplasmataceae* genomes were likely developed as adaptations to their ecological niche, specifically for “quiet” co-existence with host organisms. The *Mycoplasmas* are known to colonize their hosts with no apparent clinical manifestations, using high variability of lipoproteins to trick the host’s immune system. These are the lipoproteins that are frequently encoded by the long ORFans in *Mycoplasma* genomes, alongside with “surface protein 26-residue repeat-containing proteins” and “efflux ABC transporters”. The latter functions are also associated with the obligatory parasitic lifestyle of *Mycoplasma,* which supports our hypothesis.

Our discussion is limited to the currently sequenced and annotated prokaryotic genomes. We cannot claim that *Mycoplasmatacea* are the only group that does not obey “Lipman’s rules”. The *Anaplasma* has not yet been sufficiently investigated, however, it may potentially emerge as another group of exceptions. As sequencing costs and time continue to dropping down quickly, it is very likely that the list of exceptions will continue to grow.

## Methods

### COGs database

The Clusters of Orthologous Groups of proteins (COGs) database (http://www.ncbi.nlm.nih.gov/COG/) has been a popular tool for functional annotation since its inception in 1997, particularly widely used by the microbial genomics community. The COG database is described in detail in a series of publications [79–82]. Recently, the COG-making algorithm was improved and the COG database updated [83]; however, for the purposes of our study, we preferred to use the original COG repository ftp://ftp.ncbi.nlm.nih.gov/genomes/archive/old_genbank/Bacteria/. This choice enabled us to compare the distributions of HHP and ORFans in as many as 1484 prokaryotic genomes, since COG functional classification of the encoded proteins is one of the required descriptors of all newly sequenced prokaryotic genomes [84].

A statistical analysis was conducted in R, using built-in functions and custom scripts.

## Reviewers’ comments

### Comments by Michael Galperin (MG)

**MG.** The paper by Tatarinova and colleagues reports an interesting observation, which is worth publishing in Biology Direct. I have only two major concerns.

1. The 2002 study by Lipman and colleagues (Ref. 1 in the manuscript) has been performed before the community realized that short ORFs are in fact real and code for functional proteins or small RNA, as reviewed, for example, by Storz and colleagues (PMID: 24606146, 25475548, and 20980440), Kageyama et al. (PMID: 21729735), Landry et al. (PMID: 25795211), and many others. Please mention these reviews and discuss the bias introduced by automated genome annotation that typically ignores ORFs shorter than 50 aa (or even less than 100 aa).

Authors’ response: *The requested references were added as well as the discussion of the consequences of automatic genome annotation. However, we believe that the annotation artifacts will have only a minor influence on statistical significance of our conclusions, since it puts at disadvantage all short proteins, not only the proteins of Mycoplasma.*

**MG.** 2. The figure legend is insufficient and extremely confusing. The legends to all figures need to be expanded so that there remained no confusion as to what exactly is plotted and how these values have been calculated. Specifically,

**MG.** Fig. 1. What exactly is meant by "Abundance"?

Authors’ response: *We replaced the term “Abundance” by “Relative frequency”*

**MG.** Why is the sum of all columns in panels A-D larger than 100%?

Authors’ response: *The visual effect is due to the “stacked bars” type of the plot. We re-plotted the Fig.*1*using the “side-by-side” bars.*

**MG.** Fig. 2. Why does "Frequency" go up to 200? Is the "difference between the lengths of HHPs and ORFans, aa" calculated per genome, per species or something else?

Authors’ response: *The “Frequency” refers to the number of proteins. We expanded the legend to make the explanations clear.*

**MG.** Fig. 3. I assume that each point represents either a separate genome or a separate species. Which of the two is that?

Authors’ response: *Each point represents a separate genome.*

**MG.** Out of four yellow dots indicating Anaplasma sp., one maps very far from the others. Any explanation for that? This might reflect some inherent bias in the data that should be taken into account.

Authors’ response: *There are four Anaplasma genomes; the two in the top half of the plot infect cattle, and the outlier infects cats, dogs and horses. We added the explanation to the main document. Out of four genomes from the Anaplasma genus, A. marginale (2 strains) and A. centrale cause anaplasmosis in cattle, while A. phagocytophilum - in dogs, cats and horses. ORFans of A. marginale and A. centrale are, on average, 47 aa longer than HHPs. ORFans of A. phagocytophilum are, on average, 172 aa shorter than the HHPs. There are three times more HHPs than ORFans in A. marginale and A. centrale, while in A. phagocytophilum this ratio is equal to 1.3, while the total number of HHPs is approximately the same. Therefore, the effect can be attributed to either annotation artifact or to host specificity.*

**MG.** Also, the text says "Campylobacter" while the figure lists "Campylobacterale", a non-existent taxon.

Authors’ response: *The figure was modified.*

**MG.** Fig. 4. Same problems as in Fig. 1.

Authors’ response: *The figure was modified.*

**MG.** Also, does panel A represent a single genome of Mycoplasma genitalium (which one?) or an average of all five genomes? Same with Mycoplasma hyopneumoniae, there are three strains of it listed in Table 2.

Authors’ response: *The plot shows the histograms of protein lengths of M. genitalium (M. genitalium G37 uid57707, NC_000908) and M. hyopneumonia (M. hyopneumoniae 232 uid58205, NC_006360). We modified the legend.*

**MG.** Fig. 5. Same problems as in Fig. 2.

Authors’ response: *The “Frequency” refers to the number of proteins. We expanded the legend to make the explanations clear.*

### Comments by Vladimir Kuznetsov (VK)

**VK**. It is a common belief that the ORFan sequence length distributions are distinctly different between protein sequences with and without homologs in bacterial and archaeal genomes. Therefore, authors tested this state by a comprehensive analysis of all annotated prokaryotic genomes and focusing on certain exceptions. The results of this study meet the "novelty" by showing that Mycoplasmataceae genomes have very distinctive distributions of the ORFans lengths. The authors proposed that it might help to explain the “mysterious” long ORFans in Mycoplasmataceae. This work studied the length distributions of "having homologs proteins" (HHPs) and "non-having homologs proteins" (orphans or ORFans) in all currently annotated completely sequenced prokaryotic genomes. The authors confirmed the findings of the study by Lipman et al. (2002) showing that (i) mean of HHP lengths is longer than mean of ORFan lengths and (ii) in general, the frequency of distribution of ORFan lengths has a relatively narrow peak, whereas the HHPs are spread over a wider range of the length values. However the authors found that about thirty genomes do not follow to this rule, especially, in Mycoplasma and Ureaplasma genomes. In the both genomes, ORFans have the "atypical" sequence length frequency distributions, with the length mean of ORFans larger than the length mean of HHPs. It is a common believe that the ORFan sequence length distributions are distinctly different between protein sequences with and without homologs in bacterial and archaeal genomes. Therefore, authors tested this state by a comprehensive analysis of all annotated prokaryotic genomes and focusing on certain exceptions. The results of this study meet the "novelty" by showing that Mycoplasmataceae genomes have very distinctive distributions of the ORFans lengths. The authors proposed that it might help to explain the “mysterious” long ORFans in Mycoplasmataceae

**VK.** Major comments • Results should be statistically supported. P.6: “…the bell-shaped distribution has left tail” is confusing. The bell-shaped (normal) distribution by its definition can’t has any asymmetry and tail. Actually, the Fig. 2 exhibits an asymmetric shape and a mixture of at least two distribution function functions. Statistical tests of the normality and the mixture distribution function should be implemented and interpreted appropriately.

Authors’ response: *We agree, the distribution is indeed not normal, and we added the detailed discussion to the Additional file**1**. We performed Shapiro-Wilk test of normality, resulting in the test statistic W = 0.9507, p-value < 2.2×10*^*-16*^*, therefore indicating that the distribution is not normal. Q-Q plot (Additional file**1**: Figure S1) also supports this result. Next, we used an approach by Sahu and Cheng to investigate whether the distribution can be modeled as a mixture of two normal distributions. Weighted Kullback-Leibler distance between 2-component and 1-compoment model was found to be 0.18, which is not sufficiently large to describe the distribution as the mixture of two normal distributions.*

**VK**. Because the empirical frequency distribution functions are skewed, the parameters (mode, median, S.D. and other descriptive statistics parameters) of the studied frequency distributions should be estimated and discussed. These results may be discussed and described in the Material and Methods section (with the statistical tests, *P*-values etc.) The quantitative characteristics of length distributions should be compared and supported by statistical test’s *P*-values etc. To be reproducible, the processed data and the major result tables should be included in Additional file 1.

Authors’ response: *The focus of this paper was to validate Lipman’s hypothesis that HHPs are, on average, longer than ORFans, using the currently annotated prokaryotic genomes. We discovered that there are genomes where the lengths of HHPs and ORFans are not significantly different. As we mentioned in the text, the majority of exceptions to the Lipman's rule belong to the Mycoplasmataceae family. We added the statistical tests for the differences of mean length between ORFans and HHPs, the results are presented in the Additional file**3**: Table S2.*

**VK.** A phylogenetic tree of the genus listed in the Table 1 and specific evolution characteristics should be provided to help readers seeing the overview of the comparison.

Authors’ response: *We added the tree to the Additional file**1**.*

**VK.** The limitations of the methods, open questions of this study and future directions should be discussed.

Authors’ response: *Our discussion is limited to currently sequenced and annotated prokaryotic genomes. We cannot claim that Mycoplasmas are the only group that does not obey "Lipman's rules". Anaplasmas may appear as another such group when this family is better annotated. It is also possible that further sequencing of new genomes will expand the list of exceptions.*

**VK**. Minor comments “We calculated the Correlation Variation…”. It seems, the author wish to say “ We calculated the Coefficient Variation…”.

Authors’ response: *Thank you very much, this is, indeed, the coefficient of variation.*

**VK.** On page 17, under “List of abbreviations”, to be consistent with the main text, CG and CG3 need to be changed to GC and GC3.

Authors’ response: *The list of abbreviations is now consistent.*

**VK.** Figures 2 and 5, authors should add labels to the plot (to keep the same pattern as other figures).

Authors’ response: *The figures were modified*

### Comments by Igor Rogozin (IR)

**IR**. I think that this paper is a useful contribution to the field, I do not see any major methodological problems. It may be better to list "…possible biological explanations of this phenomenon" in the Abstract (although I am not sure about the space limitations).

Authors’ response: *We have listed several possible explanations: "We offer several possible biological explanations of this phenomenon, such as adaptation to Mycoplasmataceae’s ecological niche, specifically for “quiet” co-existence with the host organisms".*

## Additional files

Additional file 1:
**Statistical analyses and taxonomic tree reconstruction.** (DOCX 46 kb) Additional file 2: Table S1. Genomic GC content and genic GC_3_ content for annotated species of *Mycoplasma, Spiroplasma*, and *Ureaplasma*. (DOCX 22 kb) Additional file 3:
**Description of selected bacterial genomes.** Difference between COGs and ORFans. (XLSX 314 kb)

## Abbreviations

COG: A cluster of orthologous groups of genes; each COG consists of groups of proteins found to be orthologous across at least three lineages
GC content: Relative frequency of guanine and cytosine
GC_3_ content: Relative frequency of guanine and cytosine in the 3rd position of a codon
HHP: Having homologs protein; *here* COG-annotated protein
ORF: An open reading frame
ORFan: Non-HHP; *here* a protein-encoding gene that is not linked to any COG
