# Supplementary material for: The mysterious orphans of Mycoplasmataceae
Source: Biol Direct. 2016 Jan 8;11:2. doi: 10.1186/s13062-015-0104-3 (PMC4706650; doi:10.1186/s13062-015-0104-3)
Supplement: Additional file 1: — Statistical analyses and taxonomic tree reconstruction. (DOCX 46 kb) [file 13062_2015_104_MOESM1_ESM.docx]

# Additional file 1

We computed mean lengths of HHPs and ORFans for every annotated genome and analyzed distribution of differences of lengths between HPPs and ORFans. The mean of this distribution is equal to 138 amino acids, median is 141 amino acids, mode is 141 amino acids, and standard deviation is 51 amino acids. We performed Shapiro-Wilk test of normality, resulting in the test statistic W = 0.8836, p-value < 2.2×10^-16^, therefore indicating that the distribution is not normal. Q-Q plot (Supp. Figure 1) also supports this result.


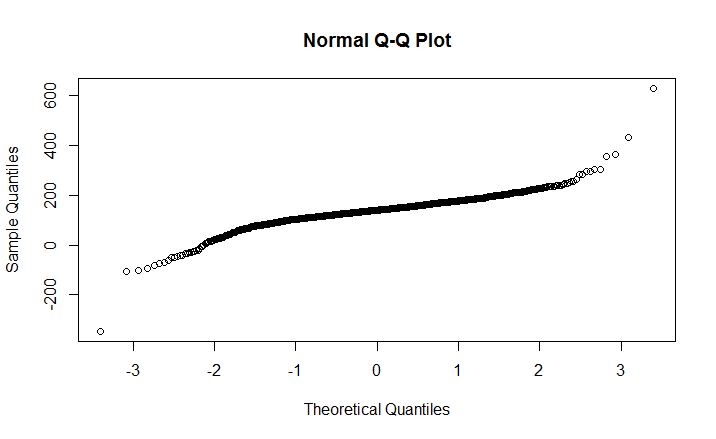


Supp. Figure 1: Q-Q plot for the differences of length between HHPs and ORFans

Next, we used an approach by Sahu and Cheng [1, 2] to investigate whether this distribution can be modeled as a mixture of two normal distributions. Weighted Kullback-Leibler distance between 2-component and 1-compoment model was found to be equal 0.18, which is not sufficiently large to describe the distribution as the mixture of two normal distributions.

For the selected genera discussed in this paper (Table 1), we constructed a taxonomic tree (Supp. Figure 2) using the “class2tree()” function from the R package “taxize”.[3]


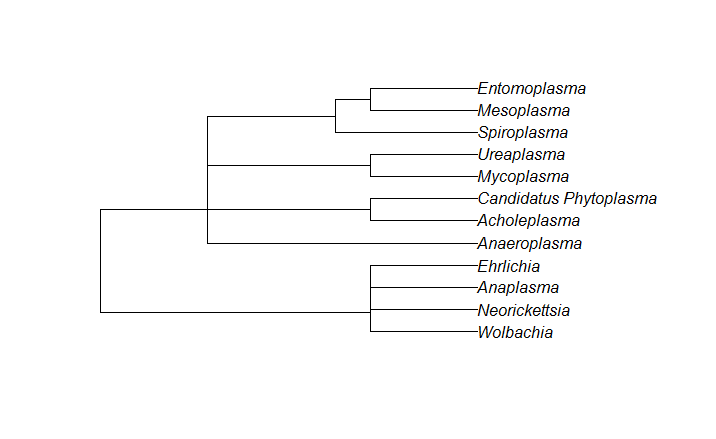


Supp. Figure 2: Taxonomic tree for the species from Table 1

**References**

1. Sahu SK, Cheng RCH. A fast distance-based approach for determining the number of components in mixtures. Canadian Journal of Statistics. 2003;31(1):3-22.

2. Tatarinova T, Bouck J, Schumitzky A. Kullback-Leibler Markov chain Monte Carlo--a new algorithm for finite mixture analysis and its application to gene expression data. Journal of bioinformatics and computational biology. 2008;6(4):727-46.

3. Chamberlain S, Szoecs E, Foster Z, Boettiger C, Ram K, Bartomeus I et al. Taxonomic Information from Around the Web. 0.6.6 ed. CRAN2015. p. Taxonomic information from around the web. This package interacts with a suite of web 'APIs' for taxonomic tasks, such as verifying species names, getting taxonomic hierarchies, and verifying name spelling.
