# Supplementary material for: The mysterious orphans of Mycoplasmataceae
Source: Biol Direct. 2016 Jan 8;11:2. doi: 10.1186/s13062-015-0104-3 (PMC4706650; doi:10.1186/s13062-015-0104-3)
Supplement: Additional file 2: Table S1. — Genomic GC content and genic GC3 content for annotated species of Mycoplasma, Spiroplasma, and Ureaplasma. (DOCX 22 kb) [file 13062_2015_104_MOESM2_ESM.docx]

Table S1: Genomic GC content and genic GC_3_ content for annotated species of *Mycoplasma, Spiroplasma*, and *Ureaplasma*.

| Organism | GC_3_ | Genomic GC content | Genome length |
| --- | --- | --- | --- |
| *Spiroplasma apis B31 uid230613* | 0.200327 | 0.283040 | 1160554 |
| *Spiroplasma chrysopicola DF 1 uid205053* | 0.168678 | 0.287990 | 1123322 |
| *Spiroplasma diminutum CUAS 1 uid212976* | 0.113885 | 0.254591 | 945296 |
| *Spiroplasma syrphidicola EA 1 uid205054* | 0.176023 | 0.292010 | 1107344 |
| *Spiroplasma taiwanense CT 1 uid212975* | 0.137940 | 0.230113 | 11138 |
| *Spiroplasma taiwanense CT 1 uid212975* | 0.104737 | 0.238734 | 1075140 |
| *Mycoplasma agalactiae PG2 uid61619* | 0.205494 | 0.297071 | 877438 |
| *Mycoplasma agalactiae uid46679* | 0.201856 | 0.296241 | 1006702 |
| *Mycoplasma arthritidis 158L3 1 uid58005* | 0.223677 | 0.307095 | 820453 |
| *Mycoplasma bovis HB0801 uid168665* | 0.204811 | 0.293079 | 991702 |
| *Mycoplasma bovis Hubei 1 uid68691* | 0.205157 | 0.292889 | 948121 |
| *Mycoplasma bovis PG45 uid60859* | 0.202470 | 0.293147 | 1003404 |
| *Mycoplasma capricolum ATCC 27343 uid58525* | 0.092527 | 0.237704 | 1010023 |
| *Mycoplasma conjunctivae uid59325* | 0.173892 | 0.284902 | 846214 |
| *Mycoplasma crocodyli MP145 uid47087* | 0.155452 | 0.269537 | 934379 |
| *Mycoplasma cynos C142 uid184824* | 0.147378 | 0.257004 | 998123 |
| *Mycoplasma fermentans JER uid53543* | 0.154001 | 0.269460 | 977524 |
| *Mycoplasma fermentans M64 uid62099* | 0.158663 | 0.268595 | 1118751 |
| *Mycoplasma fermentans PG18 uid197154* | 0.156132 | 0.268170 | 1004014 |
| *Mycoplasma gallisepticum CA06 2006 052 5 2P uid172630* | 0.246981 | 0.316319 | 976412 |
| *Mycoplasma gallisepticum F uid162001* | 0.247519 | 0.313959 | 977612 |
| *Mycoplasma gallisepticum NC06 2006 080 5 2P uid172629* | 0.248044 | 0.316202 | 938869 |
| *Mycoplasma gallisepticum NC08 2008 031 4 3P uid172631* | 0.248610 | 0.315942 | 926650 |
| *Mycoplasma gallisepticum NC95 13295 2 2P uid172625* | 0.248706 | 0.315947 | 953989 |
| *Mycoplasma gallisepticum NC96 1596 4 2P uid172626* | 0.247410 | 0.316470 | 986257 |
| *Mycoplasma gallisepticum NY01 2001 047 5 1P uid172627* | 0.247923 | 0.316050 | 965525 |
| *Mycoplasma gallisepticum R high uid161999* | 0.242985 | 0.314674 | 1012027 |
| *Mycoplasma gallisepticum R low uid57993* | 0.243143 | 0.314699 | 1012800 |
| *Mycoplasma gallisepticum S6 uid200523* | 0.246344 | 0.314669 | 985444 |
| *Mycoplasma gallisepticum VA94 7994 1 7P uid172624* | 0.248476 | 0.315846 | 964110 |
| *Mycoplasma gallisepticum WI01 2001 043 13 2P uid172628* | 0.248994 | 0.315617 | 939844 |
| *Mycoplasma genitalium G37 uid57707* | 0.235046 | 0.316891 | 580076 |
| *Mycoplasma genitalium M2288 uid173372* | 0.243793 | 0.316748 | 579558 |
| *Mycoplasma genitalium M2321 uid173373* | 0.243807 | 0.316742 | 579977 |
| *Mycoplasma genitalium M6282 uid173371* | 0.244104 | 0.316710 | 579504 |
| *Mycoplasma genitalium M6320 uid173370* | 0.244533 | 0.316767 | 579796 |
| *Mycoplasma haemocanis Illinois uid82367* | 0.299288 | 0.353336 | 919992 |
| *Mycoplasma haemofelis Langford 1 uid62461* | 0.354797 | 0.388514 | 1147259 |
| *Mycoplasma haemofelis Ohio2 uid162029* | 0.354023 | 0.388128 | 1155937 |
| *Mycoplasma hominis ATCC 23114 uid41875* | 0.165119 | 0.271182 | 665445 |
| *Mycoplasma hyopneumoniae 168 L uid205052* | 0.196551 | 0.284650 | 921093 |
| *Mycoplasma hyopneumoniae 168 uid162053* | 0.196624 | 0.284567 | 925576 |
| *Mycoplasma hyopneumoniae 232 uid58205* | 0.196548 | 0.285612 | 892758 |
| *Mycoplasma hyopneumoniae 7422 uid212968* | 0.195023 | 0.285097 | 898495 |
| *Mycoplasma hyopneumoniae 7448 uid58039* | 0.194458 | 0.284889 | 920079 |
| *Mycoplasma hyopneumoniae J uid58059* | 0.194191 | 0.285222 | 897405 |
| *Mycoplasma hyorhinis DBS 1050 uid228933* | 0.131243 | 0.259078 | 837447 |
| *Mycoplasma hyorhinis GDL 1 uid87003* | 0.130647 | 0.259062 | 837480 |
| *Mycoplasma hyorhinis HUB 1 uid51695* | 0.131508 | 0.258835 | 839615 |
| *Mycoplasma hyorhinis MCLD uid162087* | 0.132853 | 0.258765 | 829709 |
| *Mycoplasma hyorhinis SK76 uid181997* | 0.131696 | 0.258920 | 836897 |
| *Mycoplasma leachii 99 014 6 uid162031* | 0.093432 | 0.236673 | 1017232 |
| *Mycoplasma leachii PG50 uid60849* | 0.092273 | 0.237505 | 1008951 |
| *Mycoplasma mobile 163K uid58077* | 0.117239 | 0.249513 | 777079 |
| *Mycoplasma mycoides capri LC 95010 uid66189* | 0.188954 | 0.291848 | 1840 |
| *Mycoplasma mycoides capri LC 95010 uid66189* | 0.092346 | 0.238159 | 1153998 |
| *Mycoplasma mycoides SC Gladysdale uid197153* | 0.102919 | 0.239510 | 1193808 |
| *Mycoplasma mycoides SC PG1 uid58031* | 0.098171 | 0.239656 | 1211703 |
| *Mycoplasma ovis Michigan uid232247* | 0.240392 | 0.316925 | 702511 |
| *Mycoplasma parvum Indiana uid223379* | 0.160417 | 0.269751 | 564395 |
| *Mycoplasma penetrans HF 2 uid57729* | 0.134652 | 0.257175 | 1358633 |
| *Mycoplasma pneumoniae 309 uid85495* | 0.411101 | 0.399791 | 817176 |
| *Mycoplasma pneumoniae FH uid162027* | 0.403169 | 0.400037 | 811088 |
| *Mycoplasma pneumoniae M129 B7 uid185759* | 0.408628 | 0.400088 | 816373 |
| *Mycoplasma pneumoniae M129 uid57709* | 0.411421 | 0.400080 | 816394 |
| *Mycoplasma pulmonis UAB CTIP uid61569* | 0.153015 | 0.266365 | 963879 |
| *Mycoplasma putrefaciens KS1 uid72481* | 0.139499 | 0.269392 | 832603 |
| *Mycoplasma putrefaciens Mput9231 uid198525* | 0.140889 | 0.269618 | 859996 |
| *Mycoplasma suis Illinois uid61897* | 0.199426 | 0.310762 | 742431 |
| *Mycoplasma suis KI3806 uid63665* | 0.201065 | 0.310772 | 709270 |
| *Mycoplasma synoviae 53 uid58061* | 0.166114 | 0.284952 | 799476 |
| *Mycoplasma wenyonii Massachusetts uid170731* | 0.257498 | 0.339223 | 650228 |
| *Ureaplasma parvum serovar 3 ATCC 27815 uid58887* | 0.128288 | 0.254965 | 751679 |
| *Ureaplasma parvum serovar 3 ATCC 700970 uid57711* | 0.129101 | 0.254997 | 751719 |
| *Ureaplasma urealyticum serovar 10 ATCC 33699 uid59011* | 0.126853 | 0.257742 | 874478 |
